# Impact of Coronary Artery Disease on Long-Term Valve Function After the Ozaki Procedure: A Single-Center Retrospective Cohort Study (2015 – 2020)

**DOI:** 10.21470/1678-9741-2025-0187

**Published:** 2026-09-08

**Authors:** Vladlen Bazylev, Artur Mikulyak, Khadiev M. Dzhokhar, Yazan Khanfar, Vadim Karnakhin

**Affiliations:** 1 FSBI Federal Center of Cardiovascular Surgery of the Ministry of Health of the Russian Federation, Penza, Russian Federation

**Keywords:** Coronary Artery Disease, Aortic Regurgitation, Autologous, Pericardium, Propensity-Score, Matrix Metalloproteinases

## Abstract

**Introduction:**

The Ozaki procedure reconstructs the aortic valve with autologous pericardium, but long-term leaflet durability remains uncertain. Coronary artery disease (CAD) may reflect a systemic atheroinflammatory milieu associated with impaired leaflet integrity.

**Methods:**

We retrospectively studied 733 patients undergoing Ozaki reconstruction from 2015 to 2020 and assessed the association between CAD and aortic regurgitation (AR) ≥ 2 at last echocardiographic follow-up. Baseline heterogeneity was addressed using propensity-score overlap weighting based on age, sex, body mass index, body surface area, weight, diabetes, European System for Cardiac Operative Risk Evaluation II, aortic stenosis, bicuspid valve, and peripheral artery disease. Effects were estimated as weighted risk ratios (RRs) using Poisson models with robust variance. Sensitivity analyses used stabilized inverse probability of treatment weighting (IPTW) and an extended model including prior myocardial infarction/percutaneous coronary intervention.

**Results:**

Of 733 patients, 365 (49.8%) had CAD. Unadjusted AR > 2 rates were 10.1% in CAD *vs.* 5.7% in no-CAD patients (*P* = 0.037). After overlap weighting, weighted risks were 12.7% *vs.* 6.2%, yielding RR 2.05 (95% CI, 1.01 – 4.18; *P =* 0.048), with excellent covariate balance. IPTW showed consistent results: RR 2.22 (95% CI, 1.38 – 3.56; *P* = 0.001). Regurgitation-free survival was lower in CAD patients (log-rank *P =* 0.0087).

**Conclusion:**

CAD was associated with an approximately two-fold higher risk of AR ≥ 2 after Ozaki reconstruction. This association likely reflects systemic atheroinflammatory processes rather than a direct mechanical effect, supporting closer postoperative surveillance in CAD patients.

## INTRODUCTION

Aortic valve replacement remains a cornerstone of therapy for patients with aortic stenosis and other valvular pathologies. In recent years, the Ozaki procedure, an innovative procedure that reconstructs the aortic valve using autologous pericardium, has gained attention as a promising alternative to traditional valve prostheses. The approach aims to provide a more physiologic valve replacement, potentially avoiding the structural complications and lifelong anticoagulation requirements often associated with mechanical or bioprosthetic valves. Early reports have highlighted favorable hemodynamics, reduced gradients, and encouraging short-term outcomes following the Ozaki procedure.

Despite these advantages, the long-term durability of reconstructed pericardial valves remains an area of active research.

To date, the impact of CAD on valve performance after the Ozaki procedure has been largely unstudied. Understanding whether this common comorbidity contributes to worsening valve performance is critical as it may help improve patient selection, guide perioperative decision making, and inform strategies to improve leaflet durability. In this study, we aimed to determine whether CAD is associated with an increased risk of developing aortic regurgitation (AR) after the Ozaki procedure and explore potential mechanisms by which CAD-related factors may compromise autologous pericardial leaflet integrity. Our findings aim to fill a critical knowledge gap by providing valuable information to surgeons, cardiologists, and researchers seeking to improve long-term outcomes for patients undergoing this evolving surgical technique.

## METHODS

### Study Design and Patient Population

This retrospective study evaluated patients who underwent the Ozaki procedure at the Federal Center for Cardiovascular Surgery of Penza between 2015 and 2020.

#### Inclusion Criteria

- Adult patients (≥ 18 years)
- Successful Ozaki procedure
- At least one postoperative transthoracic echocardiogram (TTE)

#### Exclusion Criteria

- Previous aortic valve replacement with non-Ozaki prosthetic material
- Concomitant procedures obstructing postoperative echocardiographic evaluation
- Lost to follow-up before first postoperative TTE

### Data Collection

Demographic and clinical data were obtained from electronic medical records. Variables collected included age, sex, body mass index (BMI), presence of coronary artery disease (CAD), diabetes mellitus, left ventricular ejection fraction (LVEF), and baseline aortic valve disease (stenosis, regurgitation, or mixed disease). CAD was defined as a documented history of one or more of the following: myocardial infarction, percutaneous coronary intervention (PCI), coronary artery bypass grafting, or angiographic evidence of ≥ 50% stenosis in at least one major coronary artery.

### Surgical Technique

All patients underwent the Ozaki procedure performed by experienced cardiothoracic surgeons.The technique involved harvesting autologous pericardium, treating it with 0.6% glutaraldehyde (GA) for eight minutes, and fitting it to the aortic valve leaflets based on the patient's measured annulus dimensions. Standard protocols for connecting extracorporeal circulation and cardioplegia were followed. Concomitant coronary revascularization procedures were performed as indicated.

### Echocardiographic Evaluation

All patients underwent routine postoperative TTE before discharge and at follow-up visits. The primary endpoint of interest was the presence and degree of AR at the last available follow-up TTE. All patients underwent complex echocardiography using ultrasound devices (GE Vivid 7 Pro and GE Vivid T9, Norway). Transthoracic echocardiographic assessment of the studied parameters in patients was carried out in the heart rate range of 60 – 80/minute and after normalisation of blood pressure (< 150 and 90 mmHg). Echocardiographic measurements were performed in accordance with the recommendations of the American and European Echocardiographic Societies^[1]^. For the purposes of statistical analysis, any measurable AR beyond mild was considered “regurgitation”.

### Follow-up and Outcomes

Patients were followed at regular intervals after surgery, with clinical assessments and TTE performed at three – six months, one year, and annually thereafter. The primary outcome measure was the presence of post-Ozaki aortic valve regurgitation at the last follow-up TTE. Secondary outcome measures included progression of AR severity over time and any re-intervention related to the reconstructed valve.

### Statistical Analysis

Variables were presented as mean ± standard deviation and compared between groups using Student's *t*-test. Categorical variables were presented as frequencies (percentages) and compared using the chi-square test.

We addressed baseline heterogeneity using propensity score (PS) weighting. PS was estimated with multivariable logistic regression (treatment = CAD) using age, sex, BMI, body surface area (BSA), weight, diabetes mellitus, aortic stenosis, and European System for Cardiac Operative Risk Evaluation (EuroSCORE) II as predictors. Continuous covariates were standardized, and missing values were median-imputed. The primary analysis used overlap weighting (OW), which targets the region of greatest covariate overlap between groups. As a sensitivity analysis, we applied stabilized inverse probability of treatment weighting (IPTW) with truncation at the 99^th^ percentile to limit extreme weights. Covariate balance was assessed with standardized mean differences (SMDs), targeting < 0.10 for all covariates; we also report the weighted effective sample size (ESS). The treatment effect is reported as a risk ratio (RR) from a weighted Poisson model with robust variance, alongside weighted risks in each group.

Kaplan-Meier analysis was performed to estimate regurgitation-free survival over time stratified by CAD status. Patients were followed from the date of surgery until the event (significant regurgitation) or last follow-up.The log-ranktest was performed to compare survival curves between groups. Statistical significance was defined as *P <* 0.05. All statistical analyses were performed using R (version 4.2.2, R Foundation for Statistical Computing, Vienna, Austria).

### Ethical Considerations

The study was approved by the local ethics committee (FCCVS LEC n. 32). Written informed consent was obtained from all patients.

## RESULTS

### Study Population

A total of 733 patients who underwent the Ozaki procedure between 2015 and 2020 were included in the final analysis (Table 1). Of these, 365 patients (49.8%) had documented CAD and 368 (50.2%) did not. Patients in the CAD group were, on average, older than those without CAD (62.9 ± 8.8 *vs.* 58.1 ± 12.7 years; *P <* 0.001 ).

**Table 1:** Baseline patient characteristics.

Variable	Group without coronary heart disease (n = 368)	Group with coronary heart disease (n = 365)	*P*-value
Age (years)	58.1 ±12.7	62.9 ± 8.8	< 0.001
Men, n (%)	189(51.4%)	196(53.7%)	0.575
Height (cm)	166.0 ± 12.3	165.7 ± 12.4	0.672
Weight (kg)	79.5 ± 14.6	83.1 ± 15.3	0.004
Body surface area	1.91 ± 0.24	1.95 ± 0.25	0,012
Body mass index	28.9 ± 5.0	30.3 ± 5.5	0.003
NYHA class (intermediate)	2.19 ± 0.87	2.36 ± 0.83	0,009
Previous MI, n (%)	7(1.9%)	90 (24.7%)	< 0.001
Previous PCI, n (%)	0 (0.0%)	45(12.3%)	< 0.001
PAD, n (%)	0 (0.0%)	12(3.3%)	< 0.001
COPD, n (%)	5(1.4%)	8 (2.2%)	0.417
Pulmonary hypertension, n (%)	25 (6.8%)	36 (9.9%)	0.135
Diabetes mellitus, n (%)	56(15.2%)	99(27.1%)	< 0.001
Creatinine (µmol/l)	98.0 ± 30.1	99.0 ± 28.9	0.625
Renal failure, n (%)	21 (5.7%)	25 (6.8%)	0.558
LVEF (% of mean)	58.9 ± 9.1	59.3 ± 8.8	0.547
Aortic stenosis, n (%)	333 (90.5%)	349 (95.6%)	0,013
Aortic regurgitation, n (%)	29 (7.9%)	18(4.9%)	0.137
Bicuspid valve, n (%)	49(13.3%)	26(7.1%)	0.007
EuroSCORE II (average)	3.32 ± 1.78	3.92 ± 2.15	< 0.001

COPD=chronic obstructive pulmonary disease; EuroSCORE=European System for Cardiac Operative Risk Evaluation; LVEF=left ventricular ejection fraction; MI=myocardial infarction; NYHA=New York Heart Association; PAD=peripheral artery disease; PG=percutaneous coronary intervention

Baseline anthropometric measurements showed that patients with CAD had a slightly higher mean BSA (1.95 ± 0.25 *vs.* 1.91 ± 0.24 m^2^; *P =* 0.012) and BMI (30.3 ± 5.5 *vs.* 28.9 ± 5.0 kg/m^2^; *P =* 0.003). Comorbidities were more common in the CAD group, including a significantly higher rate of previous myocardial infarction (24.7%o *vs.* 1.9%; *P <* 0.001), previous PCI (12.3% *vs.* 0%; *P <* 0.001), and diabetes mellitus (27.1% *vs.* 15.2%; *P <* 0.001).

Measurements of cardiac structure and function revealed no significant difference in mean LVEF (*P* = 0.547). However, the incidence of aortic stenosis was higher in the CAD group (95.6%) *vs. 905%; P =* 0.013). In contrast, bicuspid aortic valves were more common in the non-CAD group (13.3% *vs.* 7.1%; *P =* 0.007). Preoperative risk assessment showed that patients with CAD had a higher mean EuroSCORE II score (3.92 ± 2.15 *vs.* 3.32 ± 1.78; *P <* 0.001).

### Propensity Score Matching with Overlap Weighting and Inverse Probability of Treatment Weighting

The primary PS model achieved excellent balance across all prespecified covariates (all post-weighting SMDs < 0.10; n = 0 covariates ≥ 0.10). The PS ranged 0.071 – 0.933 with ESS ≈ 673 in the weighted sample (Table 2). After OW on age, sex, BMI, BSA, weight, diabetes, EuroSCORE II, aortic stenosis, peripheral artery disease, and bicuspid aortic valve, the weighted risk of AR ≥ 2 was 12.68% in CAD *vs.* 6.18% in no-CAD, yielding RR 2.05 (95% confidence interval [CI] 1.01 – 4.18; *P* = 0.047) (Table 3).

**Table 2:** Propensity score (PS) weighting diagnostics.

Weighting method	N (unweighted)	Events (AR ≥ 2)	PS range	Weight median	Weight p95	Weight max.	Effective sample size	Covariates with post-weighting SMD ≥ 0.10
OW (primary)	733	60	0.071 – 0.933	0.461	0.680	0.929	673.1	0
IPTW	733	60	0.071 – 0.933	0.929	1.569	2.778	661.6	0

AR=aortic regurgitation; IPTW=inverse probability of treatment weighting; OW=overlap weighing; SMD=standardized mean difference

**Table 3:** Weighted effect estimates for aortic regurgitation ≥ 2 by coronary artery disease (CAD) status.

Weighting method	Weighted risk CAD	Weighted risk no-CAD	Risk ratio	95% CI	*P*-value
OW (primary)	12.68%	6.18%	2.05	1.01 – 4.18	0.0478
IPTW	13.86%	6.25%	2.22	1.38 – 3.56	0.0010

Cl=confidence interval; IPTW=inverse probability of treatment weighting; OW=overlap weighing

Stabilized IPTW with 99^th^-percentile truncation produced weighted risks 13.86% *vs.* 6.25% and RR 2.22 (95% CI 1.38 – 3.56; *P =* 0.0010) with all post-weighting SMDs < 0.10 and ESS ≈ 662 (Tables 2 and 3). After explicitly correcting for baseline heterogeneity with PS weighting, the association between CAD and clinically significant AR (≥ 2) persists and is directionally consistent across weighting strategies, with the OW analysis indicating approximately a twofold higher risk.

### Kaplan-Meier Survival Analysis

Kaplan–Meier survival analysis demonstrated a significant difference in regurgitation-free survival between patients with and without CAD. Specifically, patients with CAD were significantly more likely to develop significant regurgitation (grades 2 and 3) than those without. At the end of the six-year follow-up, the CAD group exhibited a significantly lower cumulative regurgitation-free survival (log-rank *P =* 0.0087) (Figure 1).

**Fig. 1:**
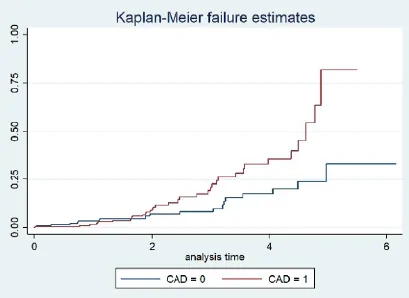
Kaplan-Meier analysis of regurgitotion-free survival by coronary artery disease (CAD) status.

Specifically, 36 events were observed in the CHD group compared with 24 in the non-CHD group.

The obtained results indicate that the presence of CAD significantly increases the risk of developing moderate or severe regurgitation after the Ozaki procedure.

## DISCUSSION

Our quantitative results highlight that the presence of CAD is associated with a higher incidence of clinically significant AR after autologous pericardial leaflet reconstruction, suggesting that patients with CAD may represent a higher-risk subgroup for long-term leaflet durability.

The Ozaki procedure reconstructs the aortic valve by fabricating leaflets from autologous pericardial tissue^[2]^. This approach is gaining popularity as an alternative to traditional aortic valve replacement due to potential advantages such as improved biocompatibility, reduced immunogenicity, and elimination of the need for lifelong anticoagulation^[3]^. However, the long-term mechanical strength of autologous pericardial leaflets remains a major challenge, and our results suggest that patients with CAD may have a less favourable biological background for sustained leaflet integrity.

### Relation of Our Results to Pathophysiology

The main question arising from our results is why patients with CAD, compared with those without CAD, have a higher incidence of postoperative regurgitation after valve reconstruction. It is known that atherosclerotic disease and CAD reflect a chronic pro-inflammatory and proteolytic milieu^[4]^ that alters not only coronary perfusion but also the biochemical and biomechanical environment of the heart. Chronic ischaemia, inflammation, and endothelial dysfunction may collectively promote enzymatic activity that degrades collagen^[4]^, the major load-bearing component of autologous pericardial valves created during the Ozaki procedure.

Pericardial tissue is a collagen-rich substrate^[5,6]^, and its collagen content and fiber organization are integral to its mechanical stability. Leaflets created by the Ozaki procedure experience cyclic mechanical loads. These include shear stress from blood flow, bending stress during valve motion, and tensile stress during valve closure^[7,8]^. Collagen fibers, as the main load-bearing components, ensure that even thin valves maintain structural integrity under these physiological demands. Mechanical stability is largely dependent on the concentration, properties, and orientation of collagen fibers^[9,10]^. When collagen fibers are damaged — either by enzymatic degradation or unregulated remodeling — the integrity of the leaflets may be compromised, which may lead to progressive regurgitation. Although observational, our data support an association between CAD and impaired valve leaflet integrity and are compatible with a pathophysiological link. Given that CAD severity correlates with various markers of adverse remodeling and may influence local factors relevant to leaflet deterioration, increased matrix metalloproteinase (MMP) activity is a plausible contributor to this association.

Although we did not measure MMP levels directly in this study, numerous previous studies have consistently demonstrated that MMP expression and activity are increased in the setting of CAD, contributing to structural degradation in many cardiovascular tissues^[11^, ^12]^.

Elevated MMP levels — particularly MMP-1, -2, -3, -7, -8, -9, -12, and -13 — have been reported in patients with CAD, notably within vulnerable atherosclerotic plaques^[11^ ^12^ ^13^ ^14^ ^15^ ^16^ ^17^ ^18]^. Elevated MMP activities including MMP-1, -2, -3, and -9 have been observed in situ, highlighting their enzymatic activity in atherosclerotic plaques. Our previous work demonstrated that in patients with CAD and chronic heart failure after myocardial infarction, MMP-9 levels correlate with indices of left ventricular (LV) remodeling^[19]^: each 1 ml increase in end-diastolic volume (EDV) increases the probability of increased MMP-9 levels by 8% (significant at EDV > 193 ml), each 1% decrease in ejection fraction (EF) increases the probability by 10% (significant at EF < 53%), and each one-point increase in the Syntax score increases the probability by 4%. These results highlight the important role of MMP-9 in regulating LV structure and function in CAD.

Additionally, MMPs have been detected in pericardial fluid, and analysis of this fluid may provide insight into the severity of ischemia or heart failure^[20^ ^21^ ^22]^. Emerging evidence suggests that MMP concentrations in pericardial fluid reflect the local cardiac environment and participate in profibrotic signaling that could drive LV remodeling in ischaemic conditions^[23]^. For example, pericardial fluid MMP-2 levels increase as LV volume increases and LV function decreases, acting as a possible autocrine/paracrine mediator influencing myocardial structure and function. Notably, the ratio of active to total MMP-2 is higher in pericardial fluid than in plasma, highlighting the localized, biologically active nature of these enzymes in the heart. In parallel, MMP-dependent gelatinolytic activity in pericardial fluid increases with LV remodeling^[22]^. MMPs degrade key extracellular matrix (ECM) components, including collagen and elastin, and therefore contribute to both physiological turnover and pathological proteolysis leading to structural deterioration^[24]^. Their involvement in bioprosthetic valve remodeling and eventual calcification has been reported, suggesting that mechanical stress and MMP-mediated degradation co-result in valve dysfunction in the long term^[25,26]^. In normal valves, MMP-2 and MMP-9 ensure ECM homeostasis, while pathological valves exhibit elevated levels of MMP-9 and novel gelatinase bands (25, 30, and 35 kDa), indicating tissue degeneration and infiltration by macrophages and activated fibroblasts^[27]^.

### MMPs in Bioprosthetic Valves and the Crosslinking Effect

It has been shown that GA-crosslinked bioprosthetic valves initially exhibit minimal MMP activity. However, explanted bio-prostheses often exhibit elevated levels of MMP-9 and activated forms of MMP-2 and MMP-9, particularly in valves with tissue rupture, whereas calcified valves exhibit lower gelatinase activity^[27]^. Other crosslinking agents such as hexamethylene diisocyanate, diphenylphosphoryl azide, or ribose also reduce MMP-mediated degradation by stabilizing collagen^[28]^. The presence of MMP activity in implanted materials is often attributed to host cell infiltration, which may lead to ECM remodeling and progressive deterioration^[27]^.

Based on these findings, an MMP-rich pericardial environment may accelerate collagen breakdown in autologous pericardial valve leaflets. Over time, such enzymatic degradation could weaken leaflet structure, leading to thinning, stretching, and malcoaptation, ultimately manifesting as progressive AR. The higher incidence of leaflet regurgitation in patients with CAD is compatible with this hypothesis and suggests that a systemic MMP-rich, atherometabolic milieu — of which CAD is one manifestation — may be detrimental to autologous leaflet integrity.

Taken together, prior studies indicate that atherosclerotic and inflammatory states upregulate MMPs (MMP-2/-9) in cardiac compartments, which can weaken collagen-rich tissues.

We did not measure MMPs or pericardial fluid in this study; therefore, these mechanistic considerations remain hypothesis-generating and warrant prospective biomarker work.

Our findings are supported not only by statistical associations but also by the time course of events: Kaplan-Meier analysis shows that differences in regurgitation-free survival between the CAD and non-CAD groups widen progressively over several years. This pattern is more consistent with a gradually progressive degenerative process than with an immediate postoperative complication and suggests chronic ECM weakening rather than a purely technical problem related to the surgical technique.

Previous studies on structural valve degeneration of surgical and transcatheter bioprostheses have linked dyslipidaemia, metabolic syndrome, systemic inflammation and overall atherosclerotic burden to accelerated leaflet calcification and failure^[29^ ^30^ ^31^ ^32]^. While CAD has typically appeared as part of this broader risk profile rather than as an isolated causal factor^[30^ ^31^ ^32^ ^33]^.

To our knowledge, no prior study has specifically examined the relationship between CAD and durability of autologous pericardial leaflets after the Ozaki procedure. Our findings therefore extend the existing literature by suggesting that patients with clinically manifest CAD may be at increased risk of late regurgitation after autologous leaflet reconstruction, in line with the concept that a systemic pro-inflammatory and proteolytic state is detrimental to valve tissue

### Limitations

This study has several limitations. First, it was a retrospective, single-center observational study; therefore, causality cannot be inferred.

Second, the mechanistic explanation involving MMP activity remains hypothesis-generating. We did not directly measure MMPs, tissue inhibitors of metalloproteinases (TIMPs), pericardial fluid biomarkers, or structural changes in explanted autologous pericardial leaflets.

## CONCLUSION

We hypothesise that increased MMP activity associated with systemic atherosclerotic and inflammatory disease, of which CAD is one clinical manifestation, may accelerate collagen degradation in autologous leaflets and contribute to leaflet failure after the Ozaki procedure. Although this relationship has not yet been fully elucidated, it highlights the potential importance of proteolytic enzymes for the long-term strength of autologous pericardial valves.

Future studies should focus on quantifying MMP levels in the pericardial fluid of patients undergoing the Ozaki procedure and correlating these measurements with long-term valve function. Studying the activity ofTIMPs and other ECM modulators will also be critical to understand the dynamic balance between tissue protection and destruction. Additionally, patient-specific factors such as systemic inflammation, metabolic status, and intrinsic differences in the quality of the harvested pericardium may influence leaflet longevity. Identification of these risk factors may inform preoperative risk stratification and facilitate targeted interventions, including pharmacological MMP modulation or pericardial tissue pretreatment, to extend the functional longevity of reconstructed aortic valves.

## Data Availability

The authors declare that the data supporting the findings of this study are available within the article.
